# User-centered design and spatially-distributed sequential electrical stimulation in cycling for individuals with paraplegia

**DOI:** 10.1186/s12984-022-01014-6

**Published:** 2022-05-08

**Authors:** Roberto S. Baptista, Marina C. C. Moreira, Lucas D. M. Pinheiro, Tiago R. Pereira, Gabriel G. Carmona, Joao P. D. Freire, Julia A. I. Bastos, Antonio Padilha Lanari Bo

**Affiliations:** 1 Laboratory of Automation and Robotics-LARA, University of Brasilia, Brasilia, Brazil; 2 Graduate Program in Biomedical Engineering, PPGEB, University of Brasilia, Brasilia, Brazil; 3 Graduate Program in Rehabilitation Sciences, PPGBCR, University of Brasilia, Brasilia, Brazil; 4 School of Information Technology and Electrical Engineering, The University of Queensland, Brisbane, Australia

**Keywords:** User-centered design, Functional electrical stimulation, FES cycling, Spatially-distributed sequential electrial stimulation, CYBATHLON

## Abstract

**Background:**

In this work, we share the enhancements made in our system to take part in the CYBATHLON 2020 Global Edition Functional Electrical Stimulation (FES) Bike Race. Among the main improvements, firstly an overhaul, an overhaul of the system and user interface developed with User-centered design principles with remote access to enable telerehabilitation. Secondly, the implementation and experimental comparison between the traditional single electrode stimulation (SES) and spatially distributed sequential stimulation (SDSS) applied for FES Cycling.

**Methods:**

We report on the main aspects of the developed system. To evaluate the user perception of the system, we applied a System Usability Scale (SUS) questionnaire. In comparing SDSS and SES, we collected data from one subject in four sessions, each simulating one race in the CYBATHLON format.

**Results:**

User perception measured with SUS indicates a positive outcome in the developed system. The SDSS trials were superior in absolute and average values to SES regarding total distance covered and velocity. We successfully competed in the CYBATHLON 2020 Global Edition, finishing in 6th position in the FES Bike Race category.

**Conclusions:**

The CYBATHLON format induced us to put the end-user in the center of our system design principle, which was well perceived. However, further improvements are required if the intention is to progress to a commercial product. FES Cycling performance in SDSS trials was superior when compared to SES trials, indicating that this technique may enable faster and possibly longer FES cycling sessions for individuals with paraplegia. More extensive studies are required to assess these aspects.

## Background

In this paper, we describe the preparation of Team EMA-Brazil to participate in the CYBATHLON 2020 Global Edition Functional Electrical Stimulation (FES) Bike Race, following our participation in the first ever CYBATHLON 2016 Edition. We will present the main improvements, with a focus on User-centered design. The main aspects presented are: improved system design and user interface, remote access to allow telerehabilitation due to the SARS-CoV-2 pandemic, and the comparison between two stimulation techniques in the context of FES Cycling: single electrode stimulation (SES) and spatially distributed sequential stimulation (SDSS).

The CYBATHLON initiative, by Swiss Federal Institute of Technology (ETH) Zurich, is a platform to advance the research in assistive technologies and shorten the distance to everyday use scenarios. A competition is held in a 4-year interval where athletes (who are called “pilots”) with motor impairment compete against each other in six race disciplines: powered arm prostheses, powered leg prostheses, powered exoskeletons, powered wheelchairs, functional electrical stimulation (FES) bikes, and brain-computer interface (BCI) [1].

In the face of the SARS-CoV-2 pandemic, the global competition scheduled for 2020 adopted a remote format, and teams competed in different time zones and locations. The CYBATHLON 2020 Global Edition occurred between 13 and 14 November 2020 with the participation of 51 teams from 20 countries. Besides the actual team races, the live-streamed event exhibited portraits and talk with experts [2].

Functional electrical stimulation is the only technique able to restore movement in paralyzed muscles which enable individuals with paraplegia to perform a pedaling movement in a recumbent bike [3, 4]. In the 2020 Global Edition Bike Race, pilots were required to cover a distance of 1200 meters in under 8 min, an increase from the previous 750 meters in the 2016 edition. Due to the remote format, the race was not held on a track, but the recumbent FES Bike was fixed to an advanced indoor bike trainer widely used by elite cyclists (KICKR Smart Trainer, Wahoo Fitness L.L.C., United Kingdom).

The SARS-CoV-2 pandemic also had a significant impact on the activities of our research group. We had to completely suspend our activities in this project for over 4 months and resumed the activities with restricted access to labs, in-person meetings, and training sessions prior to the competition.

Leading up to the CYBATHLON 2020 competition and considering the CYBATHLON initiative, we applied to our system development a User-centered design (UCD) approach. UCD is a framework that derives its approach from a range of disciplines and techniques to tie the whole design process to information about the end-users [5, 6].

To assess the resulting improvements in our system, we used the System Usability Scale (SUS), the most widely used standardized questionnaire for the assessment of perceived usability [7]. In the context of assistive devices, the SUS has been widely used as an evaluation tool for telerehabilitation systems [8] and robot-assisted devices [9].

From the engineering side, in order to gain agility in prototyping, we adopted the Robot Operating System (ROS) [10, 11] as a development tool. ROS contains a collection of tools, libraries, and conventions to facilitate the integration of distributed computers, sensors, and data exchange between complex algorithms. The system works as a peer-to-peer communication network between entities called nodes. The communication protocol can be set, including the widely used TCP and also UDP. Nodes can be written in different programming languages (such as C++ and Python) and run in separated computer units.

The SARS-CoV-2 pandemic and social distancing protocols imposed the use of telecommunications technologies in health care in general, not only for Covid related issues. This sparked the interest in the already emerging field of telerehabilitation. We seized this occasion to incorporate a remote access option in our system. The intended context for the use of this option is defined as “remote deployment” [12], where the device is shipped to the user’s home in advance and instructions can be carried out with direct web-based interaction, and each session can be remotely supervised in real-time.

The key element in FES cycling is the stimulation of paralyzed muscles [3]. In most applications of FES cycling, stimulation is applied conveying electrical waveform signals through a single pair of surface electrodes, or single electrode stimulation (SES), although implanted FES system is also an option, including team participation at both CYBATHLON editions [4].

The main challenge for FES, in general, is to increase muscle power output while delaying fatigue. To achieve this, some parameters of the electrical waveforms are varied during task execution, mainly: frequency, amplitude, and pulse width [3, 13]. In the SES approach, a single electrode is placed at the distal and another at the proximal motor point of each muscle group. The downside of this setup is that a large portion of motor units is recruited in a non-selective manner and, since the electrodes are spatially fixed, increasing stimulation intensity can only recruit a limited number of new fibers [14].

While different approaches have been proposed to mitigate this limitation, such as [15], one alternative is to spread the stimulation intensity into smaller spatially distributed electrodes. In this setup the amplitude and pulse width may be set to similar values as when using a single electrode, but the frequency is often divided by the number of smaller electrodes used. The lower frequency electrical waveform is sent to each electrode with adequate phase shift so that each smaller portion of motor units are stimulated sequentially, mimicking biological recruitment’s asynchronous nature. This approach is called spatially distributed sequential stimulation (SDSS) [13]. SDSS has been recently investigated in reducing fatigue compared to SES in spinal cord injury (SCI) individuals in isometric leg extension [16, 17] and also in dynamic knee extension task simulating FES cycling [18]. In this paper, we present a case study comparing the use of SES and SDSS in the CYBATHLON Bike Race task. To the best of our knowledge, there is no report of SDSS used in the FES Cycling task in the literature.

This paper is structured as follows: in the Methods Section we first present the Participant, next our Preparation for the CYBATHLON 2020 event, then the System Design followed by the Experimental Study. The Results Section presents the outcome of User Evaluation, the Experimental Study and our Performance at CYBATHLON 2020. In the Discussion Section we analyze the User Evaluation and System Design, our Participation at the CYBATHLON 2020 and the Experimental Study. Finally, we present closing remarks in the Conclusion Section.

## Methods

### Participant

The study was based on a single participant, also referred to as the pilot, a 42-years-old male with a trauma-induced SCI at the T9 level (7 years post-injury, ASIA Impairment Scale (AIS) grade A), selected using guidelines presented in [19]. The pilot is in good health and physical form, with no comorbidities, and engages in constant physical training as a Paralympic athlete. He uses a commercial-grade stimulation device (Dualpex 961, Ibramed, Brazil) as a tool for regular therapy, based on FES-induced muscle strengthening and isometric contractions.

The same pilot participated in previous CYBATHLON events (CYBATHLON 2016 Zurich and CYBATHLON 2020 rehearsals). Due to COVID-19 safety measures, he was the sole participant in our project post-2020. Before rehearsals, the project and the pilot were approved by a local Ethics Committee and medical check, respectively. The system was approved for the CYBATHLON competition in a remote TecCheck with an ETH representative. Under the Helsinki Declaration, the participant signed an informed consent.

### Preparation for the CYBATHLON 2020

Due to the SARS-CoV-2 pandemic, our team could not conduct training sessions from March to July 2020, when the strict lockdown was in place. The pilot relied upon FES-induced exercises at home to maintain muscle tone and health benefits during this time. Training for the CYBATHLON started in August. The training was comprised of 90-min sessions, three times a week until the first week of November 2020.

Although the user kept himself active during the lockdown period, FES cycling training was brought back to the initial conditioning stages due to the low intensity of the isometric exercise conducted at home. Hence, the training sessions after the break were stationary, initially using a trainer with no resistance. In the following weeks, the load was increased gradually to prepare the pilot for the arrival of the smart-trainer used in the competition.

Cycling distance and speed increased with training as the pilot familiarized himself with the finalized system, official setup, and the new trainer.

Similarly to our previous CYBATHLON experience [20], preparation for the FES Bike Race first targeted developing endurance. Once the fitness level enabled completing three entire races (considering the rules applied at the CYBATHLON 2020), the primary training goals shifted towards developing speed and force. The week before the event, the pilot was given a 5-day resting period to allow for muscle recovery and mental preparation.

### System design

#### Co-design approach

Our goals in refining our system from the 2016 event were: (1) to reduce the participation of engineers in training sessions; (2) give more control of the system to the end-user while ensuring safety; (3) provide the user with more detailed real-time information without being overwhelmed. We applied the following User-centered design principles, using the terminology proposed in [6].

Within this approach, we (1) conducted interviews about user perspectives after each training session to gather user perception and desire. Also, we (2) defined target users and their needs, with particular attention to improvements focused on pilot preparation to take part in the CYBATHLON 2020 Race. Furthermore, we (3) applied task analysis to understand user behavior. This effort led to the design of a user interface that provides more control and information to the user, without being overwhelming, and also features safeguards for increasing current and options for session type (e.g. warm-up, long session training, race training). Additionally, we (4) engaged in cycles of rapid prototyping where engineers participated in the training, and interviews were conducted after each session to gather user perception and desire. Finally, we (5) engaged in live prototyping, which is illustrated by the constant improvements of the system.

#### System overview and setup

**Fig. 1:**
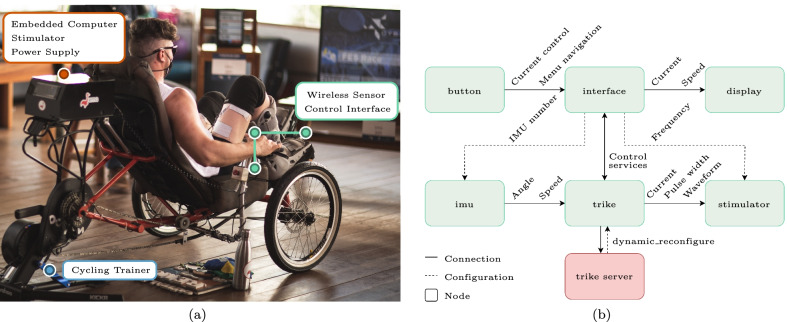
FES cycling system employed in this work. In **a**, a picture of the setup during the CYBATHLON 2020 is shown, while in **b**, a diagram of the control system is depicted

The FES cycling system employed in this work, illustrated in Fig. 1, is based on customized and commercial electronic equipment and a tricycle in a tadpole configuration (HP3 Trikes, Brazil). It was created to be used by a pilot with paraplegia due to SCI.

Besides the electronic and software improvements detailed along this article, there were two crucial mechanical improvements concerning the FES cycling system used at the CYBATHLON 2016 [3]. The first is related to the pedals and the other to the pilot’s seat. Based on feedback from other CYBATHLON 2016 teams, we have moved from the customized foot support we had built for the previous model to an orthopedic boot (Aircast, USA) attached to a cycling pedal. As for the pilot’s cockpit, we created a seat with an adjustable inclination angle and covered it with a soft and adherent material to improve comfort and decrease the pilot’s tendency to slip during ambulatory cycling. The seat’s height and inclination were adjusted during initial setup for our pilot and remained the same thereafter. With this change in the sitting position the muscle activation angles were tuned using an heuristic procedure.

Regarding the electronic hardware, the primary computing unit is an embedded system (Raspberry Pi 3, Raspberry Foundation, UK) with Ubuntu Server 18.04 as the operational system. A commercial fully programmable multichannel stimulator (RehaStim 2, Hasomed GmbH, Germany) is used, along with a wireless inertial sensor (3-Space Sensor Wireless 2.4 GHz, Yost Labs, USA) which measures the trike crank angle. A human-machine interface (with two push-buttons and a 16x2-character LCD screen display) and batteries complete the system. We designed an acrylic compartment to accommodate and protect critical electronic components from splashing water and other elements, meeting the security requirements for the CYBATHLON 2020.

The control system software was developed in modules based on the ROS platform, depicted in Fig. 1. In this architecture, each module is referred to as *node* and transmits data through channels called *topics*. So, each node is responsible for a system function such as data input, control or output. The *Button Node* transmits the ’‘button pressed’ signal to the *Interface Node*, which interprets them for menu navigation and current control when training or racing.

Next, the *Display Node* controls the LCD, which is responsible for showing the user menu during setup or the current and speed variables when racing or training. The *Interface Node* manages all communication between the control system and user (through the *Button* and *Display Nodes*), where it is possible to configure system variables. An important function is performed by the *Trike Node*. It calculates the stimulation activation moments based on the angle and speed data received from the *IMU Node* and sends the appropriate parameters (current, pulse width, and waveform) to the *Stimulator Node*.

Finally, the *Trike Server Node* is optional and runs on an external personal computer (PC) for monitoring and dynamic reconfiguration of the *Trike Node* parameters through a graphical interface built with ROS tools. An external user can use this option to track and control the session.

#### User interface

**Fig. 2:**
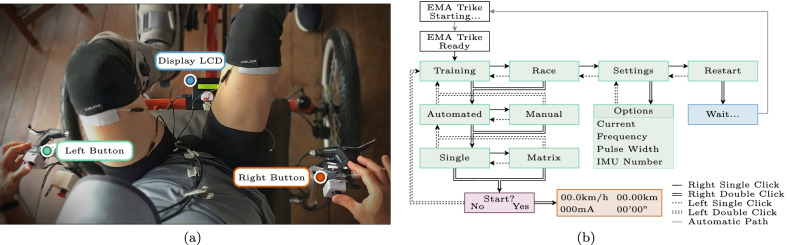
FES cycling system user interface, including **a** pilot point-of-view perspective and **b** its functional diagram

The pilot interface grants control to the pilot to operate the FES cycling system. It integrates two push-buttons and a display for real-time visualization of the system interface, as illustrated in Fig. 2.

For the CYBATHLON 2016 edition, the interface provided to the pilot was limited, displaying only stimulation intensity. The user had limited access to other control parameters. In our co-design sessions, modifications to this interface were requested. As a result, we implemented new features for the interface and reorganized visual elements presented on the display.

One of the new features was a “settings” menu that allows changes in essential parameters for a training session, such as the maximum values for stimulation pulse width, frequency, and current amplitude for the different exercise types. Another setting is the method of FES delivery: either SES or SDSS. We have also included a menu option to reset the embedded system completely. In addition to the “settings” menu, we have included menus to select major aspects for the current session. The pilot can choose if the FES intensity control is manual or automated and between a training or race session. While in the manual mode, each right button click increases the intensity, and left-click decreases it. The clicks had a latency safeguard to avoid drastic changes in current. In the automated option, a preset stimulation profile is applied, such as the ones employed in the experimental study to compare SES and SDSS. For the training session, the system starts without any stimulation applied, while the race option follows the racing guidelines for the CYBATHLON.

The pilot interface consists of screens functionally represented by the diagram in Fig. 2 and messages displayed to the pilot on the LCD. Within the figure, the white rectangles represent system startup screens and the green ones are the navigation menus or submenus for setting the exercise type and other functionalities. The purple rectangle represents the start confirmation screen, in which a question is presented to the pilot to confirm his decision. The orange one depicts the screen during an exercise in progress, designed to display the cadence, distance, elapsed time, and intensity of the stimulation current (exercise type manual training) or pulse width (exercise type automated training). Finally, the blue rectangle represents the response screen to the restart option. to display the result of a pilot’s choice.

There are other response screens in each of the parameters sub-menu in the settings menu. A message “OK” appears on the display when changing the parameter is successful or “ERROR” in case of failure.

For navigation between the set of interface screens, the two command buttons identified in Fig. 2 are used. Navigation is carried out using single and double clicks in the right and left buttons. Double-clicking the right button takes the menu to the lower level (below the current one) and the left button to the upper level (above the current one).

In the start confirmation screen, identified in purple in Fig. 2, double-clicking the right button confirms any change (“Yes” decision) and double-clicking the left button denies any change (“No” decision), thus returning to the top-level screen.

We have added maximum limits to which the pilot can change the maximum current intensity, pulse width, and frequency parameters. These limits are set according to each pilots fitness level, in our case the limits were: 100 mA, 50 Hz and 450 $\mu s$. Thus, avoiding adjustments that could harm the end-user in a training session.

During a manual training or manual race, LCD shows the exercise screen (identified as orange in Fig. 2), and the single or double right click increases the stimulation current, while the single or double left click decreases it. There is also a limit on individual increments per second for current intensity or pulse width during manual training. Each click increases or decreases 2 mA at a lower limit of 500 ms.

The “Restart” menu offers the pilot the option to completely restart the embedded system, both operating and control systems.

For pilot safety, we added the functionality to interrupt stimulation, terminate, and restart the entire control system by simultaneously clicking both buttons on any interface screen. This new safety measure is a new option similar to the emergency-power-off (EPO) button included in the commercial stimulator.

#### Remote access

Monitoring and operating the FES cycling system requires attention and experience. Applying electrical current improperly can harm the user. Therefore, using a graphical interface for viewing and modifying relevant system variables in an external PC becomes relevant for error diagnosis and prevention.

**Fig. 3:**
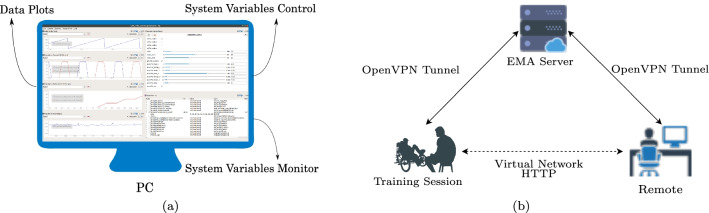
FES cycling system, including **a** PC graphical interface **b** remote access representation

We built the graphical interface depicted in Fig. 3 which plots stimulation, cadence and other data, and monitors and controls system variables. This detailed overview facilitates the interaction of researchers and health professionals in the FES cycling system development and inspection. This interface enables the visualization of plots of the stimulation and cadence data, in addition to being able to control system variables. The interface can be accessed locally on a PC connected via a USB port to the embedded system or remotely through the internet.

The remote system lets a faraway member of our team assist in a training session, viewing system data or controlling parameters, as depicted in Fig. 3. We use the OpenVPN software and ROS tools to achieve a virtual connection. The faraway member perceives a direct connection to the FES cycling system, but actually the data passes through a remote server. This configuration ensures end-to-end cryptography for safety and privacy issues.

#### Electrical stimulation

**Fig. 4:**
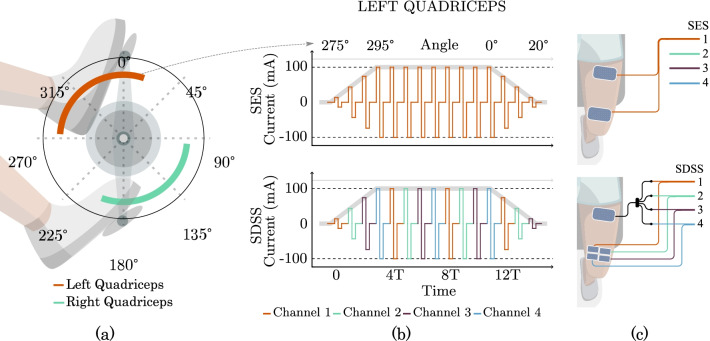
The crank angle interval for stimulation (**a**), current amplitude profile for the single electrode stimulation (SES) as well as spatially distributed sequential stimulation (SDSS) (**b**) and electrode placement for SES and SDSS (**c**).The current amplitude profile for the single electrode stimulation (SES) as well as spatially distributed sequential stimulation (SDSS) and the respective angular interval

Figure 4 displays an overview of the stimulation control scheme to generate the pedaling motion and, the different pulses profiles used and electrode placement for each case: Single Active Electrode Stimulation (SES) and Spatially Distributed Sequential Stimulation (SDSS).

In both approaches, the FES activation generates the pedaling motion, and the timing of stimuli is set by predefined crank angular position intervals. There is a shift compensation related to the crank’s angular velocity to counteract electronic and physiological delay when pedaling at different speeds. A detailed description can be found in [3, 21].

Electrical stimulation was applied using rectangular self-adhesive gel electrodes (Carcitrode, CARCI, Brazil). Pulses were rectangular, biphasic, and balanced, with a current amplitude of 100 mA. The pulse width was used as the modulation parameter, and its values were defined using prevailing literature as basis [14, 16, 18] while also slightly adapted to better suit our pilot.

Stimuli were created using a communication mode available in the stimulator called “Science Mode”, which acts as a developer tool, allowing commands to be sent from a line of code through a serial port to the stimulator. In Science Mode, the researcher can alter parameters, such as: current amplitude, frequency, and pulse width.

Among the ways to send commands to the stimulator through Science Mode, we used the single-pulse mode to generate individual stimuli with a determined channel, current amplitude, and pulse width. However, it was still necessary to create a way to repeatedly send the commands to the stimulator with the chosen frequency and the proper channel to maintain the sequence. This is done inside *Trike Node* in Fig. 1, which controls the frequency of pulses and sends commands to the stimulator. This ROS node was designed to be generic and can be used in other contexts, enabling the modification of the channels used, changing the frequency, pulse width, and current amplitude.

Regarding electrode placement, depicted in Fig. 4, for SES two 9 cm by 5 cm electrodes were applied to the rectus femoris motor points: the proximal as a reference and the distal as an active electrode. This setup is the same used in sessions for isometric contractions for this muscle group, which usually precedes the use of FES Cycling. It is expected that the physiotherapist is familiar with this electrode placement. For SDSS, we used a single 9 cm by 5 cm proximal electrode and a set of four electrodes, with 4.5 cm by 2.5 cm, forming a distal matrix of electrodes. The sizing decision was made to maintain total dimensions and preserve placement for the active electrode, i.e. the matrix in SDSS should occupy the same place as the single electrode in SES.

For SES, electrical pulses operated at a frequency of 48 Hz. For SDSS, a total of four stimulation channels were used, each at 12 Hz, matching the total of 48 Hz in SES. Sequential stimuli at a fraction of the SES frequency were chosen to maintain the overall aspect of the SES wave as illustrated in Fig. 4. SDSS requires a 4-step sequential stimulation to different electrodes; however, the stimulator does not allow sequential pulses to be sent from a single channel.

We generated time-phased pulses with externally controlled frequency for different active electrodes, using four distinct channels to resolve the issues. Finally, to preserve a single reference electrode, the team opted to create a 4 by 1 cable adapter. The strategy was feasible was feasible since none of the channels are simultaneously activated during the experiments. This setup is similar to [17] and [18].

### Experimental study

#### Protocol

In addition to the user evaluation, a study designed to compare the performance of FES cycling when applying SES or SDSS was conducted in this work. For that purpose, four sessions were dedicated to collecting data, with a minimum of 24 h of muscle recovery time between sessions.

At the start of each session, electrodes were placed and the pilot was positioned on the trike, which maintained the same overall mechanical configuration as the competition, except for the gears. The trike has 2 gears in the crank and 8 in the wheel cassette. Prior to this protocol the gears setting was chosen by the pilot and remained fixed during all sessions. Each session then consisted of (1) 5 min of cycling warm-up with manual assistance and no stimulation, (2) 15 min of FES cycling warm-up with manual assistance (if needed), (3) 10 min resting, (4) 5 min of cycling warm-up with manual assistance and no stimulation, and, finally, (5) one race following CYBATHLON rules, with a total duration of 8 min and 30 s.

In all parts of each session, the end of assistance was determined solely by the assistant, who was responsible to feel when the pilot could sustain the pedaling movement. Special attention was given in (5) not to exceed the allowed 30 s of initial assistance.

**Fig. 5:**
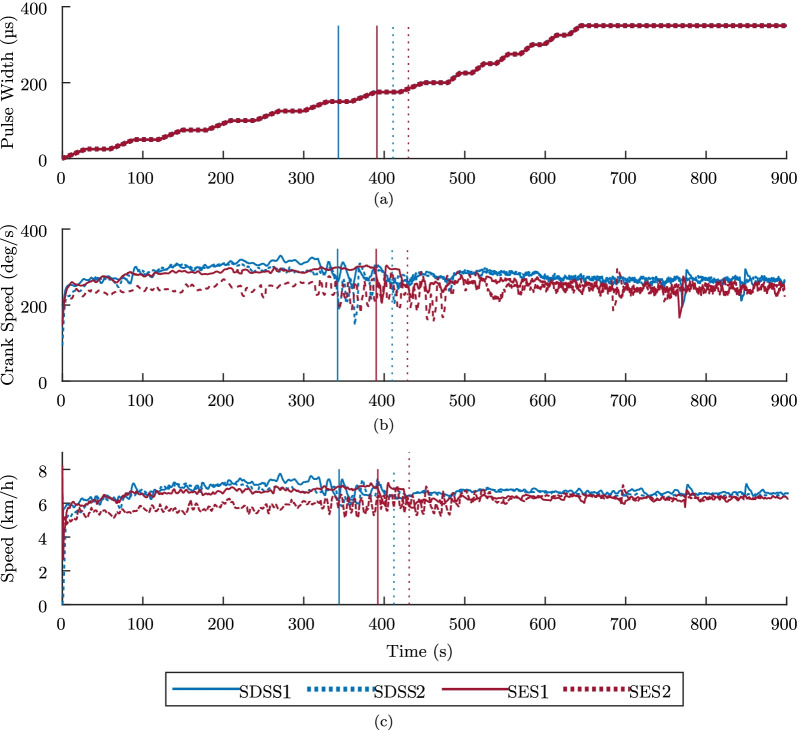
Experimental data from the “warm up” stages, i.e., stage (2), in an experimental study to compare SES and SDSS. **a** illustrate the pre-programmed FES applied, and **b** and **c** depict crank speed and trike speed, respectively. The vertical lines indicate the moment when manual assistance was removed

**Fig. 6:**
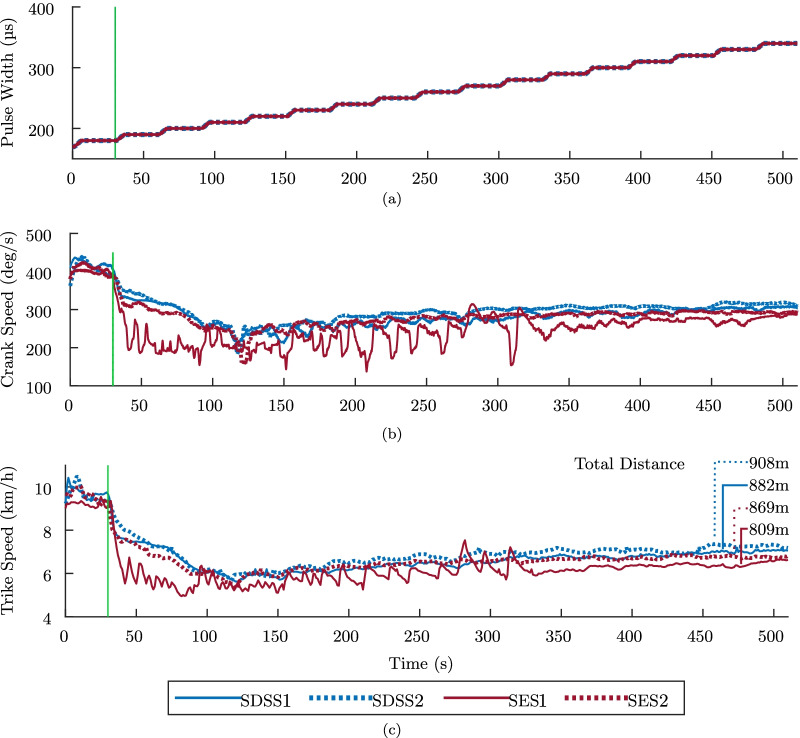
Experimental data from the “race” stages, i.e., stage (5), in an experimental study to compare SES and SDSS. **a** illustrate the pre-programmed FES applied, and **b** and **c** depict crank speed and trike speed, respectively. The vertical lines indicate when manual assistance was removed, which occurred 30 s after race start in all trials. The total distance without manual assistance is also depicted

In (1), a specialist observed muscle response, particularly to detect muscle spasms and resistance, which could indicate contractures. In the following stage, we increased the pulses duration width from 0 $\mu$s up to 350 $\mu$s, in pre-programmed increments (illustrated in Fig. 5). Finally, we followed the CYBATHLON rules in (5), where FES cycling with manual assistance (if needed) was applied for the initial 30 s, and then the participant pedaled independently for another 8 min. In (5), the pulse width started at 170 $\mu$s and saturated at 340 $\mu$s, gradually increasing in 10 $\mu$s every 25 s through ramps of 5 s, depicted in Fig. 6. Also, a maximum of 3 manual interventions were allowed during this stage. This intervention is allowed by CYBATHLON guidelines, and its purpose was simply to maintain movement continuity and avoid prolonged muscle contractions in an eventual stop, which could harm the pilot.

A single method, i.e., SES or SDSS, was applied in each session. The choice of FES parameters was based on studies where SDSS was applied on different tasks, such as [22] and our previous work [17]. Pulses with an amplitude of 100 mA were applied, and the pulse width was used as the modulation parameter. When SES was used, pulses operated at a frequency of 48 Hz. Two 9 cm by 5 cm electrodes were applied to the RF motor points. If the SDSS was employed, we used five individual electrodes: one 9 cm by 5 cm electrode placed proximally and four 4.5 cm by 2.5 cm electrodes arranged as a distal matrix. The size and distribution of distal electrodes were selected to maintain the total surface area and preserve the placement for the 9 cm by 5 cm distal electrode. In order to enable the SDSS mode, a total of four stimulation channels were used, each at 12 Hz. Sequential stimuli at a fraction of the SES frequency were chosen to maintain the overall aspect of the SES wave, as illustrated in Fig. 4.

#### Data acquisition

To compare SES and SDSS, we have mainly applied measures available from the smart trainer. Considering that several groups worldwide use the same platform, one additional motivation is that our results may be easily compared with the performance obtained in local setups. From this perspective, the following measures of cycling performance were used: time until independent cycling (i.e., time until the participant started pedaling without manual assistance), distance independently covered, and speed profile when pedaling independently.

Collected data was imported, plotted, and analyzed using a Matlab (Mathworks, USA) script. Furthermore, we also used readings from the IMU located on the crank. Regarding crank speed, the data was further filtered for improving graphical visualization. Since the output data from the smart trainer was not integrated into our customized ROS-based control software, offline synchronization was employed.

#### User evaluation

The System Usability Scale (SUS) [23] is a standardized questionnaire to assess a system’s overall usability. It consists of 10 statements evaluated by the user on a scale from 1 to 5 (strongly disagree, disagree, neutral, agree, strongly agree). Brooke et al. [23] disclose the usability statements, as well as describes score contributions to each question. The final (SUS) score ranges from 0 to 100, a full hundred indicating the best user perception.

In this work, the SUS questionnaire was filled by the same pilot who co-designed the system and took part in the experimental study. Since the participant was both co-designing and evaluating the system, results from the SUS questionnaire must be analyzed in this context. Nevertheless, the participant was instructed to provide the most sincere answer to the statements presented in the questionnaire.

## Results

### User evaluation

Table 1 shows the queries and the corresponding results obtained when applying the SUS questionnaire. The participant described a high likelihood to use the system frequently; also thought the system was easy to use; found the functions in the system well-integrated and intuitive; and demonstrated confidence in using the system (all positive aspects rated 5, on the scale). Nevertheless, the pilot found the system complex (4, on the scale); reported a high likelihood of needing support from a technical person (4, on the scale); and neither agreed or disagreed that he had to learn many things before using the system (3, on the scale). The results also indicate the pilot has observed few inconsistencies during training sessions (2, on the scale) and has evaluated this is definitely not awkward to use (1, on the scale). The total SUS score was 77.5.

**Table 1:** Questions and scores obtained from user evaluation using the SUS questionnaire

	Item	Scale
1	I think that I would like to use this system frequently	5
2	I found the system unnecessarily complex	4
3	I thought the system was easy to use	5
4	I think that I would need the support of a technical person to be able to use this system	4
5	I found the various functions in this system were well integrated	5
6	I thought there was too much inconsistency in this system	2
7	I would imagine that most people would learn to use this system very quickly	5
8	I found the system very awkward to use	1
9	I felt very confident using the system	5
10	I needed to learn a lot of things before I could get going with this system	3
	SUS Score	77.5

### Experimental study

Figures 5 and 6 present the collected speed data in all four sessions, along with the corresponding pulse width. Both figures illustrate the PW-modulation stimulation intensity, which is gradually increased, as well as crank speed and trike speed in each four completed sessions. Vertical lines in both speed plots indicate when manual assistance was no longer allowed. Hence, for the “race” condition, in all trials, FES cycling without any manual assistance started at 30 s.

Table 2 lists the calculated performance measures employed to analyze each session. The values were calculated using data from the smart trainer. Within the “warm up” stage, average results show that the required assistance decreased from 410.5 s (when using SES) to 377.0 s (when using SDSS), an 8.2% decrease. The pilot was also able to pedal longer distances using the distributed stimulation, on average 953.5 m, an increase of 12.3% compared to the 849.1 m using conventional stimulation. The speed in “warm up” was also higher when using SDSS, averaging 6.63 km/h, while SES sessions provided an average of 6.21 km/h.

Concerning results from “race” stages, using SDSS allowed the pilot to reach longer distances, an average of 895.2 m against 839.9 m obtained when using SES, an increase of 6.59%. Average speed improved 6.39$\%$ when comparing averages of 6.91 km/h in SDSS and 6.50 km/h in SES. Finally, comparing results obtained in different stages, standard deviation during “race” was higher than that obtained during “warm up”.

### Performance at CYBATHLON 2020

At the CYBATHLON FES Bike Race, the system performed as designed. In the CYBATHLON 2020 Global Edition each team had to setup a local competition hub with the support of the event organizers. Due to the extra effort in partially hosting the event and the novelty of the SDSS setup for the pilot and team, we opted to take a conservative choice and compete using the SES configuration. This decision was taken mainly to avoid shortcomings in the system setup during the event. The pilot fully controlled the stimuli magnitude and no incidents occurred. Based on the pilot’s performance in training, it was expected the pilot would complete the first two runs, while in the third trial, effects of fatigue would be likely visible. As anticipated, the pilot achieved 944 m and 966 m in the first two attempts, while the last run ended at 7 min and 10 s and 815 m. The last run was shorter due to the pilot touching his leg for the third time, the maximum allowed according to the event rules. The 966 m mark granted Team EMA sixth place in the FES Bike Race.

The 966m mark at 8 min results in an average speed of 7.25 km/h, which is higher than the best result in the protocol, achieve in SDSS$_2$, presented in Table 2. However, in the protocol the stimulation profile was preset with constant increments in intensity and peaking at 100 mA and 340 $\mu s$. During the race our pilot controlled the intensity at his will and reached a peak of 100 mA and 450 $\mu s$.

**Table 2:** FES cycling performance measures from experimental study to compare SES and SDSS. Sub-indexes 1 and 2 refer to solid and dashed data depicted in Figs. 5 and 6, respectively

	Warm up				Race			
Session	SES$_1$	SES$_2$	SDSS$_1$	SDSS$_2$	SES$_1$	SES$_2$	SDSS$_1$	SDSS$_2$
End of assistance (s)	391	430	343	411	30	30	30	30
Distance without assistance (m)	880.4	817.8	1,005.8	901.1	809.9	869.8	882.1	908.3
Speed without assistance, Avg (km/h)	6.01	6.42	6.56	6.71	6.27	6.72	6.80	7.02
Speed without assistance, Std Dev (km/h)	0.41	0.33	0.44	0.35	0.90	0.83	0.88	0.49

## Discussion

### User evaluation and system performance

Since its creation, Project EMA has employed co-design strategies to develop technological resources dedicated to individuals with SCI. In this process, the user’s experience and feedback are vital to define initial requirements and further improve system usability and performance. Nevertheless, our recent effort to update the platform used in the 2016 edition [3] was constrained due to restrictions caused by the SARS-CoV-2 pandemic. In our particular case, social distancing measures prevented meetings and demonstrations that were crucial for development purposes.

Nevertheless, as a result of joint efforts from developers, physiotherapist, and the pilot, the user approved the mechanical, electronic, and software improvements in the final system, as indicated by Table 1. The table also illustrates that, despite being well acquainted with FES, the overall system has technical components that the user has very little knowledge of. Indeed, while technical improvements implemented by the team (e.g., ROS-based system, remote access) are regarded as important achievements to the project sustainability, the user indicated the system is complex, and possibly requiring support from a technical person to operate. Despite not critical in terms of safety, these issues raised by the participant definitely affects the user experience and the system usability.

Recalling the User-centered design principles and co-design approach to meet the goals in refining our system from the 2016 event we can highlight the main contributions of each stakeholder to the final system configuration. The pilot’s key inputs were reflected in: (1) the new seat leading to a comfortable sitting position that also favored the transfer movement in and out of the trike; (2) the user interface with full control of the stimulation parameters, giving him a sense of empowerment and synergy with the device, although the navigation through the multiple menus was confusing; (3) the user interface with reduced information in training or racing, the user requested that only key numbers were displayed during practice, which allowed us to keep the small LCD screen display. The therapist’s key inputs were reflected in: (1) user interface and PC interface with maximum boundary setting for stimulation according to the pilot’s fitness level. Special attention was given to provide certain freedom for the pilot, while ensuring his safety; (2) user interface and PC interface which easily adjusts the stimulation parameters at the beginning of a session to keep up with the improvement of the pilot’s fitness level; (3) PC interface with and overview and reports of the session, to track the pilot’s progress and adapt stimulation intensity. The engineers’ key inputs were mainly reflected in: (1) the PC interface which provides an overview of each variable representing sensor readings, control signals and communication. This resulted in a powerful tool for rapid testing and diagnosis; (2) ROS based system with easily re-configurable software and hardware setup and straightforward everyday use. The adoption of ROS was fundamental when incorporating the live prototyping approach, while keeping the system fully functional. This was particularly evident when designing and executing the SES and SDSS experimental protocol

One additional aspect that was assessed in interviews based on open questions involved the system safety and comfort. Indeed, while being adapted from the same commercial tricycle used in 2016, our current system was further updated to enable seat height and posture adjustments and firmer leg placements to ensure optimal pedaling and efficient force transfer. Cycling ergonomy is important not only to improve performance but also in terms of safety, ensuring the user’s physical integrity. Safety of bionic technologies for individuals with SCI have to be held to a high standard, most often surpassing standards required for equivalent medical devices often found in the market. Our support team composed of health specialists ascertained that the improvements were safe. At the end, there were no safety incidents during the CYBATHLON preparations/event or the collection of SES and SDSS data.

### Participation at the CYBATHLON 2020

The Global Edition was not only a competition, an opportunity to showcase the improvements made between 2016 and 2020, or a chance to compare technologies. It also served as inspiration for those living with a disability and many others. SARS-CoV-2 came with challenges to human health and a scenario of uncertainty and isolation.

The experience showed the importance of working together, collaborating with the event organizers and teams that should have been our competitors. The overall feeling was one of cooperation, and can only be favorable to the development of assistive technologies and the improvement  of the lives of persons living with disabilities.

### Experimental study

#### Overall findings

Team EMA designed a protocol of comparison between single electrode stimulation SES and SDSS in the context of FES cycling. Although there have been studies showing the superiority of SDSS to SES, these were based on alternative protocols, such as knee extension in isometric testbeds. To the best of our knowledge, this was the first work where SES and SDSS were compared in FES cycling for individuals with paraplegia.

In the “warm up” and “race” phases, the results indicated a better performance when using SDSS. The improvement is signaled by a lower intensity to maintain movement, a higher speed with fewer variations, and hence the ability to travel longer distances. Interestingly, crank and trike speed maintain roughly the same profile, suggesting that IMUs are valid sensors for such studies. The IMU size and portability endorse its use for ambulatory cycling.

The outcome of our experiments was that, indeed, SDSS performed better, which could be the result of sequential stimulation being slightly more akin to a voluntary contraction. In naturally occurring contractions, the central nervous system CNS sends stimuli to a selection of muscle fiber at a time; the asynchronous recruitment of each sub-group allows for fibers to contract and relax, delaying fatigue. FES contractions work differently. Transcutaneous electrodes are faced with the challenge of traversing dermis and adipose tissue before reaching motor points, diminishing selectivity. Moreover, traditional FES often stimulates a multitude of fibers simultaneously, wearing the muscle. Nevertheless, the following sections further explore potential explanations for the different results obtained using each method.

#### Electrode placement and topology

FES is conventionally applied through a pair of electrodes over each muscle to be stimulated; this technique is referred to in this paper as SES. In SDSS, as implemented in this work, one of the electrodes from SES is substituted by a matrix. Each element of the matrix is activated at a different instant to mimic biological recruitment’s asynchronous nature.

One should note that there are multiple options in terms of organization and placement of the matrix and the order of electrode activation in SDSS. As depicted in the “Methods” portion of this document, we opted for a 2-by-2 aggregated arrangement (instead of distributed) to be activated sequentially from the distal-lateral to proximal-medial electrodes.

For all SDSS experiments in the present work, the team used the matrix of electrodes in the distal rectus femoris (RF) motor point, as in [22]. The corresponding paper concluded that the force integral did not differ between distally or proximally-placed matrix.

The matrix used in this work comprised four 4.5 cm by 2.5 cm electrodes to preserve roughly the same electrode placement and geometry as in the SES protocol. It is noteworthy that a 1 cm separation was left between each matrix element to avoid current bleeding. Other authors also employ this topology [13, 24, 25].

The findings of both literature and this work indicate lower levels of induced fatigue using SDSS. However, in our study, which is limited in sample size and number of sessions, the numerical gains were shyer compared to other works. One of the possible reasons for this is that our matrix tries to preserve the size and placement of SES distal electrode. Indeed, distributing the matrix in the muscle’s distal motor points has led to higher gains when compared to the technique of SES placement [18, 22, 26–28]. Nonetheless, the protocol involving distributed SDSS may be often time-consuming, and only suitable to be applied to large muscles, which are disadvantages of the method employed in this work.

#### Type of muscle fiber

There are two main types of muscle fiber in the human body, each with characteristics that make them more appropriate for different activities. Type I is known for its slow-setting fatigue and endurance characteristics; type II is marked by rapid fatigue but quick and powerful movements. Individuals with SCI, however, have been reported to have a higher proportion of type II than non-disabled people [29, 30].

One of the morphological differences between type I and II muscle fibers is their myosin chains. In persons with SCI, changes in myosin chains seem to occur that effectively transform a type I into type II fiber, accounting for persons with SCI having a preponderance of fast-twitch fibers. The conversion process is reported to initiate between 1 [31] up to 7 months [32] after the injury. Currently, there are no definite indications that FES can reverse or delay the loss of fast-twitch fibers. However, studies often follow patients for a maximum of twelve weeks, and longer-term effects are yet to be explored [33–35].

The patient portrayed in this text is seven years post-injury and started using FES as a therapeutic tool shortly after the first year of SCI. The injury-time leads us to believe that the pilot has a predominance of type II muscle fibers. The latter being more prone to fatigue and responsible for more forceful contractions, should be partially responsible for FES contractions behaving differently from natural contractions.

In [18], Laubacher and co-authors refer to the recruitment of type I fibers as a possible reason for the superior outcome when using SDSS. Indeed, three out of the four subjects in the concerned study featured younger injuries (6 months or less), which is possibly not enough time for fiber conversion to alter quadriceps cytological properties significantly. That being the case, these three subjects should have approximately a 40/60 ratio of type I/II fibers, and fiber selection could have played a role. The fourth subject was 24 months from SCI and should have a higher proportion of fast-twitch fibers. If fiber selection had a primordial role in SDSS, the fourth subject’s comparative results should have been inferior to his counterparts; however, the reported findings showed otherwise. Thus, fiber type selection may not be the most important factor contributing to better outcomes achieved by SDSS.

#### SDSS in FES cycling

In addition to the influence of SDSS matrix topology/placement and the type of muscle fiber in the overall performance of the asynchronous stimulation, another reason for the limited improvement in SDSS-based FES cycling may lie in the exercise itself. Indeed, the literature cited so far compares SES and SDSS in the context of isometric [13, 17, 22, 24–28, 36–43] and isokinetic exercises [18, 22, 25–27, 41]. Studies comparing SES and SDSS in isometric protocols have reported the more substantial gains in favor of SDSS when compared with isokinetic exercises. The result could be explained since the stimulated muscle does not change length; therefore, its motor points remain in the same position, a condition where SES might produce its worst performance.

In [26], Bergquist and co-authors present a unique protocol based on isometric and isokinetic exercises in the same study. The authors used the same subjects and conducted the data collection/processing using similar equipment/techniques, creating an ideal scenario for analyzing SDSS performance regarding exercise complexity. In their results, SES showed a 26% decrease from initial torque for the isometric exercises, while SDSS had a 2% decrease. In the isokinetic exercises, SES showed a 42% decrease from initial torque, while SDSS had a 7% decrease. Throughout their findings, SDSS torque, fatigue, and variability feature better results the more uncomplicated the exercise. Based on previous results from isometric studies, we may hypothesize that it is not SDSS relative performance that deteriorate in dynamic exercises, but instead SES relative performance that improves in such dynamic movements.

The present work explores SDSS in a complex motion. Cycling is a functional task with a joint activity; the stimulated muscle constantly changes lengths, muscle tension changes according to the pedaling phase, and speed is not constant. In this context, the nature of the exercise may explain why cycling featured more modest outcomes when comparing SES to SDSS.

Finally, it is also important to note that “warm up” protocols were a 15 min exercise and “race” had an 8 min and 30 s run. In contrast, isometric/isokinetic studies use a set of 20 [26] up to 200 [27] contractions, a much slimmer exercise duration.

## Conclusions

This paper presented the improvements in our system leading up to the CYBATHLON 2020 Global Edition FES Bike Race. The User-centered design approach resulted in a generally well-accepted system by the user, which was expected. However, it is not yet perceived as a system the user can use without technical personnel’s support. This indicates that we are still dealing with an experimental device, and special attention should be taken to this aspect when transitioning to a commercial device. The remote access option enables online monitoring in a global context where telerehabilitation is expected to increase.

We showcased the application of SDSS in the challenging task of an FES Bike Race with an experienced user. The results indicate that SDSS leads to performance improvements compared to SES, although further investigation is required. Our approach to developing this technology and the research context was shaped by the CYBATHLON format, which prompted us to focus on usability issues that would probably be overlooked in an engineering or clinical research. Our pilot has become a key player in our research team and an enthusiast of the FES Cycling technology.

## Data Availability

Please contact author for data requests.

## Abbreviations

FES: Functional electrical stimulation
SES: Single electrode stimulation
SDSS: Spatially distributed sequential stimulation
BCI: Brain computer interface
UCD: User-centered design
SUS: System usability scale
ROS: Robotic operating system
PC: Personal computer
SCI: Spinal cord injury
LCD: Liquid cristal display
IMU: Inertial measurement unit
VPN: Virtual private network
RF: Rectus femoris
CNS: Central nervous system
